# Effectiveness of Self-Efficacy–Based Interventions to Improve eHealth Literacy Among Community-Dwelling Older Adults: Protocol for a Systematic Review

**DOI:** 10.2196/89598

**Published:** 2026-07-09

**Authors:** Runa Tokunaga, Ayano Ando, Erika Ota

**Affiliations:** 1 Department of Nursing, Faculty of Human Sciences, Sophia University, 4-16-11, Shimoochiai, Shinjuku-ku,Tokyo, 161-8550, Japan, 81 070-8477-2795; 2 Department of Nursing, Faculty of Medical Technology, Teikyo University, Tokyo, Japan; 3 Graduate School of Nursing Science, St. Luke's International University, Tokyo, Japan

**Keywords:** eHealth literacy, older adults, self-efficacy, community-dwelling older adults, digital health, health education, systematic review, randomized controlled trial, aging

## Abstract

**Background:**

eHealth literacy is essential for enabling older adults to access, evaluate, and use digital health information effectively. Self-efficacy, defined as confidence in one’s ability to perform specific behaviors, is a key determinant of sustained health behavior change and has been widely applied in health education interventions. However, the effectiveness of self-efficacy–based interventions in improving eHealth literacy among community-dwelling older adults has not been systematically evaluated using high-quality evidence.

**Objective:**

This systematic review aims to evaluate whether self-efficacy–based interventions aimed at improving eHealth literacy among community-dwelling older adults are effective in improving health-related outcomes, such as eHealth literacy and self-efficacy.

**Methods:**

Following PRISMA-P (Preferred Reporting Items for Systematic Review and Meta-Analysis Protocols) guidelines, this review will include randomized controlled trials and cluster randomized controlled trials targeting community-dwelling adults aged ≥50 years, in which self-efficacy serves as the core theoretical framework. MEDLINE, the Cochrane Library, Embase, CINAHL, ClinicalTrials.gov, and International Clinical Trials Registry Platform will be searched without language or publication year limits. The primary outcome will be improvement in eHealth literacy measured using validated instruments such as the eHealth Literacy Scale, the eHealth Literacy Questionnaire, and the Digital Health Literacy Instrument. Secondary outcomes include self-efficacy, health behaviors, quality of life, and frailty. Two reviewers will independently and in duplicate screen studies, extract data, and assess risk of bias using the Cochrane Risk of Bias 2 tool. Where feasible, a meta-analysis using standardized mean differences will be conducted.

**Results:**

The review was registered in PROSPERO on September 7, 2025 (CRD420251139882). Literature screening, data extraction, and analysis are scheduled to be conducted between September 2025 and September 2026. The results will be synthesized using the Grading of Recommendations Assessment, Development and Evaluation (GRADE) approach and presented in summary of findings tables.

**Conclusions:**

This review will clarify the effectiveness of self-efficacy–based interventions in enhancing eHealth literacy among older adults, thereby supporting the development of evidence-informed digital health education for this population.

## Introduction

### Background

With advancements in information technology, health-, medical-, and welfare-related information is now widely disseminated through the internet and social networking services. Consequently, health literacy—the ability to select, understand, and appropriately use health information—has become increasingly important [1-3]. Among its various forms, eHealth literacy—the ability to select, evaluate, and use health information via digital media, such as the internet, social networking service, and information and communication technology tools—has emerged as a key concept in maintaining personal health in contemporary society [4].

### Prior Work

Norman and Skinner [5] defined eHealth literacy as “the ability to seek, find, understand, and appraise health information from electronic sources and apply the knowledge gained to addressing or solving a health problem,” integrating multiple literacies, including traditional health literacy, information literacy, scientific literacy, media literacy, and digital literacy.

High levels of eHealth literacy contribute to the appropriate use of medical resources, promote healthy lifestyles, and facilitate self-management [6]. As populations age, enhancing eHealth literacy among older adults has become an increasingly important public health priority.

Despite its importance, significant disparities exist in eHealth literacy levels among older adults. As global aging accelerates [7], many older individuals face challenges in adapting to an increasingly digitalized society [8]. Compared with younger populations, older adults are generally less familiar with information and communication technology tools and may lack the skills needed to evaluate and use digital information effectively. Moreover, age-related declines in vision, hearing, and cognitive function can further hinder the acquisition and practice of eHealth literacy [9]. These challenges are particularly significant among community-dwelling older adults, where access to health care and welfare services often varies by region [10].

In response, numerous interventions have been developed globally to improve eHealth literacy. Previous studies have reported that theory-based digital health education interventions may improve older adults’ ability to access, evaluate, and use online health information effectively. According to Bandura’s theory, self-efficacy can be strengthened through mastery experiences, vicarious experiences, verbal persuasion, and management of physiological and emotional states [11]. These concepts have been widely incorporated into health education and behavioral interventions targeting older adults. However, their effectiveness, theoretical foundations, and feasibility vary considerably. Pourrazavi et al [12] reviewed theory-based interventions and reported that self-efficacy was the most frequently adopted theoretical framework. Self-efficacy, defined as an individual’s belief in their ability to perform specific tasks [11], has been widely validated as an effective driver of health behavior change [13]. A systematic review by Kim and Jeon [14] found a strong association between self-efficacy and eHealth literacy, suggesting that self-efficacy–enhancing interventions may lead to more sustainable behavioral changes than purely knowledge-based approaches.

Moreover, recent studies have highlighted the mediating roles of self-efficacy and self-care in promoting health behaviors through improved eHealth literacy. For example, Wang et al [15] reported that higher eHealth literacy among community-dwelling older adults was associated with improved health-promoting behaviors, mediated by self-efficacy and self-care ability. Similarly, Wong et al [16] demonstrated that eHealth literacy and perceived social support predicted self-care behaviors among homebound older adults. A systematic review by Hua et al [17] further identified multiple individual and contextual factors influencing eHealth literacy, underscoring its complexity and multidimensional nature.

**Figure 1.**
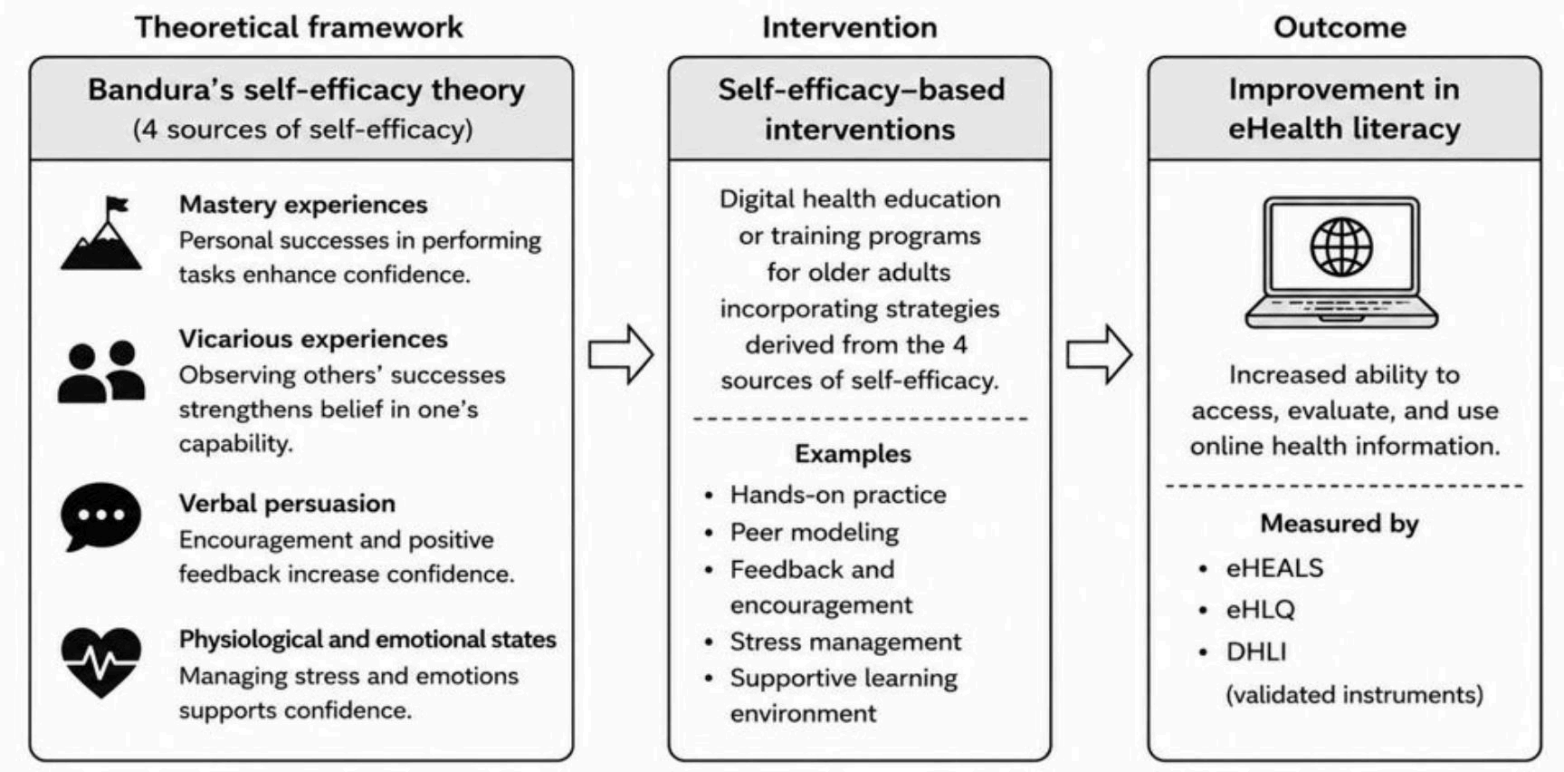
Conceptual framework of self-efficacy–based interventions for improving eHealth literacy among community-dwelling older adults. Note: this framework illustrates that self-efficacy theory [11] serves as the theoretical foundation for intervention design, and the primary expected outcome is improvement in eHealth literacy among community-dwelling older adults. DHLI: Digital Health Literacy Instrument; eHEALS: eHealth Literacy Scale; eHLQ: eHealth Literacy Questionnaire.

Despite these insights, empirical studies on self-efficacy–based interventions remain fragmented, with considerable heterogeneity in intervention content, evaluation measures, and study populations. Moreover, systematic reviews focusing exclusively on high-quality designs, such as randomized controlled trials (RCTs) and cluster RCTs, are lacking. Addressing this gap is important for generating evidence to support future eHealth literacy interventions and related practice development. The conceptual framework of this systematic review is presented in Figure 1.

### Study Objectives

Therefore, in this systematic review, we will focus on RCTs and cluster RCTs evaluating the effectiveness of interventions designed to improve eHealth literacy based on the theory of self-efficacy among community-dwelling older adults. The review will address the following research questions: (1) Are self-efficacy–based interventions effective in improving eHealth literacy among community-dwelling older adults? and (2) What characteristics of these interventions are associated with improved eHealth literacy outcomes?

Our aim is to present the protocol for a systematic review conducted in accordance with the PRISMA-P (Preferred Reporting Items for Systematic Review and Meta-Analysis Protocols) guidelines [18]. In this review, we will evaluate whether interventions aimed at improving eHealth literacy among community-dwelling older adults, based on the theory of self-efficacy, are effective in improving health-related outcomes, such as eHealth literacy and self-efficacy.

## Methods

### Registration

This systematic review protocol follows the PRISMA-P 2015 guidelines and has been registered in the PROSPERO database (CRD420251139882).

### Type of Study Design

This systematic review will include RCTs and cluster RCTs. Nonrandomized studies, including quasi-RCTs, will be excluded. Eligible studies will be selected based on the Cochrane criteria and definitions for RCTs and clinical trials [19]. This decision was made to ensure methodological rigor and consistency in accordance with Cochrane recommendations. The protocol is reported in accordance with the PRISMA-P 2015 checklist [20].

### Type of Participants

The participants will be community-dwelling individuals aged ≥50 years, regardless of sex, region of residence, or cultural background. In this review, the target population was defined as adults aged ≥50 years. Although “older adults” are often defined as those aged ≥65 years, many previous studies on eHealth literacy interventions included participants aged 50 years and older. As digital health disparities tend to emerge from this age, adopting a threshold of 50 years enables a broader and more relevant evidence base while maintaining the focus on older populations.

### Type of Interventions

In this review, we will evaluate self-efficacy–based intervention programs designed to improve eHealth literacy among community-dwelling older adults aged ≥50 years. There will be no restrictions on the setting, facilitators, implementation methods, or media used for the interventions. All types of intervention programs will be eligible for inclusion.

Interventions will be classified as self-efficacy–based if they meet at least one of the following criteria: (1) self-efficacy is explicitly identified as a theoretical framework guiding the intervention design or (2) the intervention includes components intended to enhance self-efficacy. To further characterize intervention content, components will be coded according to Bandura’s 4 sources of self-efficacy: mastery experience, vicarious experience, verbal persuasion, and physiological and affective states [11]. Interventions based on multiple theoretical frameworks will be included if self-efficacy constitutes a central or explicitly targeted component of the intervention. Studies examining self-efficacy solely as a mediator or moderator of intervention effects, without self-efficacy informing the intervention design itself, will be excluded.

The control group will include one of the following: (1) no intervention, (2) standard intervention for community-dwelling older adults, or (3) interventions not based on self-efficacy theory.

### Type of Outcome Measures

#### Primary Outcome

The primary outcome will be eHealth literacy among community-dwelling older adults, assessed using validated measurement tools. These include, but are not limited to, the eHealth Literacy Scale, the eHealth Literacy Questionnaire, and the Digital Health Literacy Instrument, which capture both perceived and performance-based aspects of eHealth literacy.

When different measurement scales are used across studies, standardized mean differences with 95% CIs will be calculated to enable comparison and synthesis of results.

#### Secondary Outcomes

The secondary outcomes to be evaluated in this review include the following:

- Self-efficacy measured using tools such as the General Self-Efficacy Scale [21].
- Health behaviors assessed using instruments such as the Health Behavior Questionnaire.
- Subjective health perception measured using tools such as the Subjective Well-Being Inventory.
- Quality of life assessed using scales such as the EuroQol 5-Dimension Questionnaire or Short Form 6-Dimension Health Utility Index.
- Frailty assessed using tools such as the Modified Frailty Index.

Other measures may be independently developed by researchers and may include outcomes such as appropriate service use or complication rates.

#### Time Frames for Outcome Measures

Changes in the primary outcome will be assessed immediately after the intervention and at follow-up assessments, including short- to mid-term follow-up periods such as 1 to 3 months after the intervention. Where available, follow-up outcomes assessed at different time points will also be considered. This allows for the evaluation of immediate and mid- to long-term effects. The secondary outcomes will be assessed on the same schedule. If studies do not assess outcomes at multiple time points, single-point data will be used for evaluation.

### Electronic Searches

This review will follow the PRISMA (Preferred Reporting Items for Systematic Reviews and Meta-Analyses) statement [18]. The databases to be searched include MEDLINE via PubMed, Cochrane Library, Embase, and CINAHL Ultimate. In addition, trial registries such as ClinicalTrials.gov and the World Health Organization’s International Clinical Trials Registry Platform will be searched to identify ongoing or unpublished studies. The search strategy will incorporate terms related to eHealth literacy, self-efficacy, and older adults. No restrictions will be placed on publication date or language, and all relevant studies reported to date will be included. A preliminary search was conducted in MEDLINE, CINAHL, Embase, and the Cochrane Library to confirm the feasibility of the review question and the availability of relevant studies addressing self-efficacy–based interventions for improving eHealth literacy among community-dwelling older adults. The preliminary search identified 2476 records, of which 1607 remained after duplicate removal. Following title and abstract screening, at least 3 studies were identified as potentially eligible for inclusion, supporting the feasibility of the planned review.

The comprehensive search strategy was developed in accordance with the PRISMA-S (Preferred Reporting Items for Systematic reviews and Meta-Analyses literature search extension) guidelines and the Peer Review of Electronic Search Strategies statement [22]. Detailed search terms, Boolean operators, and database-specific search strategies are provided in Multimedia Appendix 1. A research librarian with expertise in systematic review searches will assist in refining the strategy, using Medical Subject Headings (MeSH), where applicable. Additionally, a professor with expertise in systematic review methodology provided guidance on the review design and protocol development. The reporting of the search strategy will follow the PRISMA-S guidelines to ensure transparency and reproducibility.

### Data Collection and Analysis

Eligibility criteria will be determined in advance using the population, intervention, comparison, and outcome framework, and their relevance will be reviewed by 2 research team members. In this review, the components are defined as follows:

- Population: studies including community-dwelling adults aged ≥50 years.
- Intervention: studies must clearly indicate that (1) self-efficacy is used as the theoretical framework and (2) the primary purpose of the intervention is to improve eHealth literacy.
- Comparison: studies must include 1 of the following as a comparison group: (1) no intervention, (2) standard interventions for community-dwelling older adults, or (3) interventions not based on the theory of self-efficacy.
- Outcomes: studies must report the effect of interventions based on self-efficacy on the improvement of eHealth literacy. Studies that target core components of eHealth literacy, such as information literacy, media literacy, and computer literacy, will also be included.
- Setting: no restrictions will be placed on intervention setting; all types, including clinical-based, internet-based, telephone-based, individual, and group interventions, will be considered.
- Study design: only RCTs, including cluster RCTs, will be included.
- Publication date: no restrictions will be placed on publication date; all studies published before the search date will be included.

The exclusion criteria for this review are as follows:

- Publication type: non-peer-reviewed materials such as nonacademic magazines or reports will generally be excluded. However, unpublished RCTs identified through trial registries will be considered if sufficient methodological and outcome data are available.
- Intervention content: studies will be excluded if they (1) refer to self-efficacy but do not discuss or use it in the intervention design or (2) do not provide empirical evidence supporting the effectiveness of the intervention.

### Data Extraction and Management

Two reviewers will independently and in duplicate screen the titles and abstracts of studies identified by the search strategy and select those that meet the inclusion criteria. The full texts of the selected studies will be obtained and screened independently by the same reviewers using the same eligibility criteria. Any discrepancies will be resolved through discussion until consensus is reached.

Both reviewers will independently extract all data in duplicates to ensure accuracy. Data extraction will include (1) outcomes; (2) authors, year of publication, and country; (3) number of participants and their demographics (age and sex); (4) intervention details (design and materials); and (5) outcome measures.

The results will be summarized in a detailed summary of findings table.

### Assessment of Risk of Bias in the Included Studies

Two reviewers will independently assess the risk of bias according to the Cochrane Risk of Bias 2 tool, following the guidelines outlined in the Cochrane Handbook for Systematic Reviews of Interventions. The following types of bias will be considered: (1) selection bias, (2) performance bias, (3) attrition bias, (4) reporting bias, and (5) other sources of bias.

Disagreements will be resolved through discussion. Where possible, a meta-analysis will be conducted. The 2 reviewers will independently assess studies to be included in the meta-analysis. Any disagreements will be resolved through discussion.

### Measures of Treatment Effect

When outcome measures and scales are consistent across studies, mean differences with 95% CIs will be calculated for continuous outcomes. For dichotomous outcomes, relative risk and 95% CIs will be reported. For cluster RCTs, clustering effects will be accounted for in the analysis. Where appropriate, effect estimates will be adjusted using the reported intracluster correlation coefficient. If the intracluster correlation coefficient is not reported, we will attempt to obtain it from the study authors or use estimates from similar studies. If quantitative synthesis is not possible owing to heterogeneity in populations, interventions, or outcomes, a narrative summary will be provided, detailing population characteristics, intervention types, outcome types, and effects.

### Dealing With Missing Data

For included studies, the level of missing data for primary outcomes will be assessed using sensitivity analysis. Where possible, corresponding authors will be contacted to obtain missing data. Statistical analyses will follow a modified intention-to-treat approach wherever feasible.

### Assessment of Heterogeneity

Heterogeneity will be assessed using the chi-square test and *I*² statistic. A *P* value <.10 on the chi-square test and an *I*² value >50% will be interpreted as evidence of significant heterogeneity.

A random-effects model will be used as the default approach for meta-analysis. If heterogeneity is negligible and the included studies are sufficiently comparable, a fixed-effect model may be applied. Substantial heterogeneity alone will not automatically preclude quantitative synthesis. The decision to conduct meta-analysis will be based on both statistical heterogeneity and the clinical and methodological comparability of the included studies, including similarities in participants, interventions, outcome measures, and timing of assessment. When quantitative synthesis is not appropriate because of substantial clinical or methodological heterogeneity or insufficient data, a narrative synthesis will be provided, summarizing population characteristics, intervention details, outcome types, and effects. Where appropriate and feasible, alternative random-effects approaches, such as the Hartung-Knapp-Sidik-Jonkman method and prediction intervals, will also be considered to improve interpretation of heterogeneity and uncertainty.

### Assessment of Reporting Bias

If more than 10 studies are included in the meta-analysis, publication bias will be assessed using funnel plots and visually interpreted for asymmetry. In addition, reporting bias will be examined using appropriate statistical tests, such as the Egger test or Begg test, depending on the suitability of the data.

### Data Synthesis

Where studies are sufficiently homogeneous in terms of participants, interventions, and outcome measures, a statistical meta-analysis will be conducted using RevMan Web (Cochrane Collaboration). If quantitative synthesis is not appropriate because of substantial heterogeneity or insufficient data, the findings will be synthesized narratively. To address research question 2, intervention characteristics associated with improved eHealth literacy outcomes will be extracted and summarized descriptively. Intervention components will be categorized according to Bandura’s 4 sources of self-efficacy, and similarities and differences across interventions will be explored through narrative synthesis [11]. Where sufficient data are available, subgroup analyses based on intervention characteristics will also be considered.

### Subgroup Analysis and Assessment of Heterogeneity

Where data are available and necessary, subgroup analyses will be conducted on the primary outcomes to explore sources of heterogeneity.

If appropriate, subgroup analyses will be conducted based on characteristics, such as the type of intervention (online, face-to-face, or hybrid formats) and duration of the intervention (short-term interventions lasting a few weeks or long-term interventions lasting several months). Where data are available, subgroup analyses will also be conducted based on age categories (50‐64, ≥65, and ≥75 years) to explore potential differences in intervention effects across age groups. This approach will help in exploring factors that may influence the effectiveness of interventions aimed at improving eHealth literacy.

### Sensitivity Analysis

Sensitivity analyses will be performed. In particular, sensitivity analyses excluding studies involving participants aged 50 to 64 years will be conducted, where feasible. Studies judged to have a high risk of bias in allocation concealment or with incomplete outcome data will be excluded if they significantly impact the primary outcome.

### Summary of Findings

The quality of evidence across studies will be assessed using the Grading of Recommendations Assessment, Development and Evaluation (GRADE) approach. Each outcome will be evaluated based on study limitations, inconsistency, imprecision, indirectness, and publication bias. The quality of evidence will be rated as high, moderate, low, or very low. Results will be summarized using the GRADE summary of findings tables.

### Ethical Considerations

This study is a systematic review protocol that does not involve the collection of primary data from human participants. Therefore, ethics approval or informed consent was not required.

## Results

This review protocol was registered in PROSPERO (CRD420251139882) on September 7, 2025. Literature screening, data extraction, and quality assessment are scheduled to be conducted between September 2025 and September 2026. Data synthesis and preparation of the final manuscript are planned following completion of the review process. The findings will be synthesized using the GRADE approach and presented in summary of findings tables, where appropriate.

## Discussion

### Overview

By synthesizing the evidence across diverse educational, psychological, and behavioral interventions, the review aims to contribute to the development of evidence-informed approaches for supporting eHealth literacy among community-dwelling older adults. Previous reviews have highlighted the importance of theory-based eHealth literacy interventions and identified self-efficacy as one of the most frequently used theoretical frameworks [12]. In addition, a previous review reported a positive association between self-efficacy and eHealth literacy among older adults [14]. Building on these findings, this review will specifically focus on self-efficacy–based interventions evaluated using RCT and cluster RCT designs, thereby addressing an important gap in the existing literature. In rapidly aging societies, such as Japan, these findings may provide useful insights for future community-based digital health education and support strategies.

### Principal Findings

This systematic review will synthesize high-quality evidence on self-efficacy–based interventions targeting eHealth literacy among older adults.

### Strengths and Limitations

A major strength of this review is its focus on RCTs and cluster RCTs, ensuring a high level of methodological rigor. Furthermore, the use of the self-efficacy theory as the conceptual framework provides a solid theoretical basis for understanding mechanisms of intervention effectiveness.

However, by focusing exclusively on published peer-reviewed studies, potentially relevant gray literature can be excluded, which may increase the risk of publication bias. In addition, heterogeneity in intervention content, outcome measures, and follow-up periods may influence the comparability and interpretation of findings. Finally, the review is limited to community-dwelling older adults, and the findings may not be generalizable to institutionalized populations. To address these limitations, subgroup analyses and narrative synthesis will be conducted, where appropriate.

### Recommendations for Further Research

Future studies should explore standardized intervention protocols that can be adapted to different cultural and health care contexts. Longitudinal studies are needed to assess the sustained impact of eHealth literacy interventions over time. Additionally, further research is required to understand mediating factors, such as digital access, cognitive function, and social support, that influence improvements in eHealth literacy outcomes.

### Implications for Policy and Practice

The findings of this review are expected to inform policymakers, educators, and health care professionals in designing and implementing evidence-based eHealth literacy programs for older adults. Integrating eHealth literacy interventions into community health promotion strategies may support self-management and informed decision-making among older adults. These insights may inform future digital health strategies and educational approaches aimed at supporting older adults’ access to online health resources.

### Relevance for Clinical Practice

This review will provide clinicians and nurses with evidence-based guidance on effective strategies to enhance eHealth literacy among adults aged ≥50 years. By identifying intervention components that successfully improve digital health skills and self-efficacy, the findings will support the development of patient education programs that enable older adults to access, evaluate, and apply online health information more effectively. Improved eHealth literacy has the potential to promote better self-management, strengthen treatment adherence, and reduce health disparities in aging populations. In addition, the review will offer practical insights for integrating digital health support into routine clinical practice and for reinforcing multidisciplinary approaches to patient-centered care in an increasingly digitalized health care environment.

### Conclusions

In this systematic review protocol, we outline the methodology for evaluating the effectiveness of interventions aimed at improving eHealth literacy among community-dwelling older adults based on the theory of self-efficacy. By synthesizing evidence from RCTs and cluster RCTs, this review may clarify the characteristics of effective interventions and identify knowledge gaps in the existing literature. The findings will provide a foundation for the development of evidence-informed educational programs and inform future clinical and community-based strategies to promote eHealth literacy among older adults.

## Supplementary material

10.2196/89598 Multimedia Appendix 1 PubMed search strategy.

## Abbreviations

GRADE: Grading of Recommendations Assessment, Development and Evaluation
MeSH: Medical Subject Headings
PRISMA: Preferred Reporting Items for Systematic reviews and Meta-Analyses
PRISMA-P: Preferred Reporting Items for Systematic Review and Meta-Analysis Protocols
PRISMA-S: Preferred Reporting Items for Systematic reviews and Meta-Analyses literature search extension
RCT: randomized controlled trial
